# Kinetics of the [4+2] cycloaddition of cyclopentadiene with (*E*)-2-aryl-1-cyano-1-nitroethenes

**DOI:** 10.1007/s00706-012-0735-3

**Published:** 2012-03-02

**Authors:** Radomir Jasiński, Magdalena Kwiatkowska, Andrzej Barański

**Affiliations:** Institute of Organic Chemistry and Technology, Cracow University of Technology, Warszawska 24, 31-155 Cracow, Poland

**Keywords:** [4+2] Cycloaddition, Nitroalkene, Kinetics, Mechanism

## Abstract

**Abstract:**

The electrophilicity of (*E*)-2-aryl-1-cyano-1-nitroethenes is not sufficient to induce a zwitterionic course of their [4+2] cycloaddition to cyclopentadiene. The one-step mechanism of these reactions is indicated by the activation parameters, and the substituent and solvent effects on the reaction.

**Graphical Abstract:**

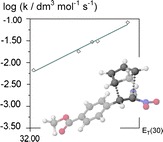

## Introduction

For the last decade, reactions of conjugated nitroalkenes have aroused our considerable interest [1–9], and we have exerted considerable effort to examine their reactivity in thermally allowed cycloaddition reactions. Especially the [4+2] cycloaddition reactions of cyclopentadiene (**1**) with strong electrophilic (*E*)-2-aryl-1-cyano-1-nitroethenes **2a**–**2g** were investigated [4–6, 9].

By means of HPLC and NOESY experiments [6], it has been unequivocally established that this reaction leads to the corresponding 6-*endo*-aryl-5-*endo*-cyano-5-*exo*-nitronorbornenes **3a**–**3g** and 6-*exo*-aryl-5-*exo*-cyano-5-*endo*-nitronorbornenes **4a**–**4g** as the only reaction products. The stereospecific reaction course suggests that, independently of the nitroalkene electrophilicity, this reaction occurs according to a concerted mechanism (paths A and B). However, stereospecificity cannot be regarded as sufficient evidence of the concertedness [10–12]. Nevertheless, it cannot be ruled out that the reaction proceeds by a zwitterionic mechanism [10, 13–15] (paths C and D), while preserving the original nitroalkene configuration in cycloadducts, when the activation barrier for the carbocyclic ring closure in postulated intermediates is lower than the corresponding barrier for rotation around the C–C bond of the cyanonitroethyl moiety. The aim of this work was to evaluate the nature of the transition complexes and to confirm or exclude the presence of zwitterionic intermediates on the reaction paths. For this purpose, the kinetics of the title reaction were investigated, and the substituent and solvent effects as well as activation parameters were determined.

## Results and discussion

**Scheme 1:**
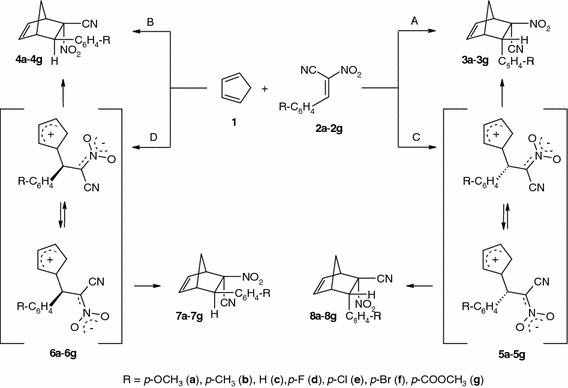

**Fig. 1:**
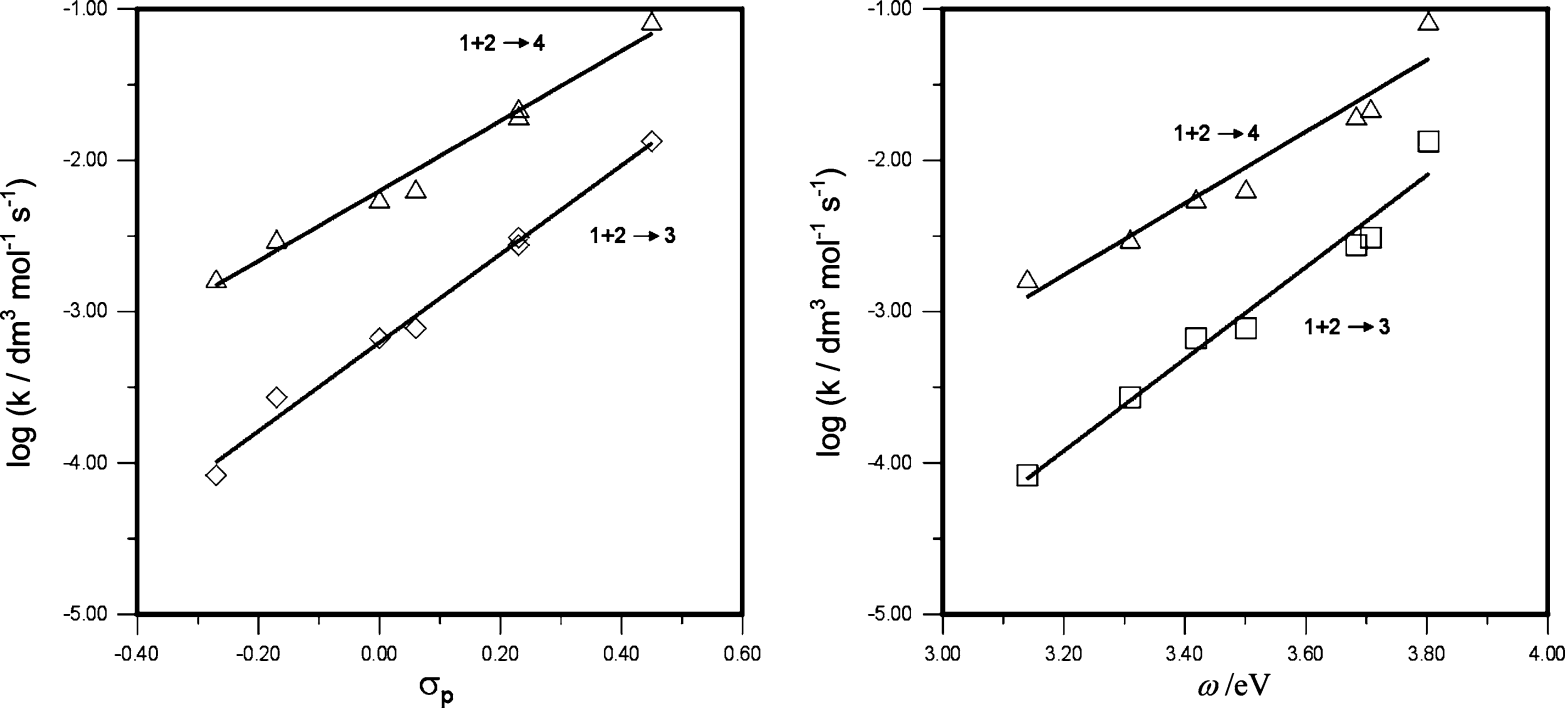
Plot of log *k* versus Hammett constants σ_p_ and global electrophilicity index ω for the [4+2] cycloaddition of cyclopentadiene with (*E*)-2-aryl-1-cyano-1-nitroethenes **2a**–**2g** (at 25 °C in nitromethane)

Kinetic studies (Table 1) showed that the rate constants of the reactions leading to the nitronorbornenes **3a**–**3g** and **4a**–**4g** (Scheme 1) increase with the nitroalkene electrophilicity. In particular, the rate constants *k*_A_ and *k*_B_ for the reactions involving the least electrophilic (*E*)-2-(*p*-methoxyphenyl)-1-cyano-1-nitroethene (**2a**) (ω = 3.14 eV [5]) are 0.08 × 10^−3^ dm^3^ mol^−1^ s^−1^ and 1.66 × 10^−3^ dm^3^ mol^−1^ s^−1^, while those for the reactions with the most electrophilic (*E*)-2-(*p*-methoxycarbonylphenyl)-1-cyano-1-nitroethene (**2g**) (ω = 3.80 eV [5]) are 13.35 × 10^−3^ dm^3^ mol^−1^ s^−1^ and 83.42 × 10^−3^ dm^3^ mol^−1^ s^−1^, respectively. We investigated the substituent effect on the reaction using classical correlation analysis, seeking the best match between substrate reactivity (log *k*_A_ and log *k*_B_) and the values of the Hammett constants σ^+^, σ_p_, σ_R_, σ_I_ [16] of the substituents. The best linear correlations (*R* = 0.995 for the reaction **1** **+** **2** → **3** and 0.991 for the reaction **1** **+** **2** → **4**) were obtained with the Hammett constants σ_p_ (Fig. 1). This means that the substituent effect is transferred to the reaction centers via both induction and resonance [17], and the transition complexes are similar regardless of the nitroalkene electrophilicity. These relationships are expressed by the Hammett Eqs. 1 and 2.

1 $$ \log k_{\text{A}} = 2.93 \cdot \sigma_{\text{p}} - 3.20\quad (R = 0.995) $$

2 $$ \log k_{\text{B}} = 2.31 \cdot \sigma_{\text{p}} - 2.20\quad (R = 0.991) $$

**Table 1:** Results of kinetic measurements of [4+2] cycloaddition of cyclopentadiene with (*E*)-2-aryl-1-cyano-1-nitroethenes **2a**–**2g**

Nitroalkene				Solvent [*Ε*_Τ_(30)]	*T*/°C	*k*_total_ × 10^3^/dm^3^ mol^−1^ s^−1^	Isomer ratio γ [**3**]/[**4**]	*k*_A_ × 10^3^/dm^3^ mol^−1^ s^−1^	*k*_B_ × 10^3^/dm^3^ mol^−1^ s^−1^	*R*	SD
Nr	R	σ_p_	ω/eV								
**2a**	OCH_3_	−0.27	3.14	Nitromethane (46.3)	25	1.74	0.05	0.08	1.66	0.998	0.01
**2b**	CH_3_	−0.17	3.31	Nitromethane (46.3)	25	3.28	0.09	0.27	3.01	0.998	0.02
**2c**	H	0.00	3.42	Nitromethane (46.3)	25	6.23	0.12	0.67	5.56	0.998	0.02
**2d**	F	0.06	3.50	Nitromethane (46.3)	25	7.26	0.12	0.78	6.48	0.997	0.03
**2e**	Cl	0.23	3.68	Nitromethane (46.3)	25	22.46	0.14	2.76	19.70	0.998	0.03
**2f**	Br	0.23	3.71	Nitromethane (46.3)	25	25.13	0.14	3.09	22.04	0.998	0.04
**2g**	COOCH_3_	0.45	3.80	Nitromethane (46.3)	5	35.15	0.15	4.58	30.57	0.998	0.05
**2g**	COOCH_3_	0.45	3.80	Nitromethane (46.3)	15	59.99	0.15	7.82	52.17	0.998	0.05
**2g**	COOCH_3_	0.45	3.80	Nitromethane (46.3)	25	96.77	0.16	13.35	83.42	0.997	0.07
**2g**	COOCH_3_	0.45	3.80	Dichloroethane (41.9)	25	34.46	0.12	3.69	30.77	0.999	0.04
**2g**	COOCH_3_	0.45	3.80	Dichloromethane (41.1)	25	33.23	0.12	3.56	29.67	0.999	0.05
**2g**	COOCH_3_	0.45	3.80	Chloroform (39.1)	5	6.26	0.10	0.57	5.69	0.999	0.03
**2g**	COOCH_3_	0.45	3.80	Chloroform (39.1)	15	10.05	0.11	1.00	9.05	0.997	0.05
**2g**	COOCH_3_	0.45	3.80	Chloroform (39.1)	25	20.28	0.12	2.17	18.11	0.999	0.04
**2g**	COOCH_3_	0.45	3.80	Tetrachloromethane (32.5)	25	7.45	0.11	0.74	6.71	0.997	0.07

It is noteworthy that the values of the reaction constants ρ in Eqs. 1 and 2 (2.93 for the reaction **1** **+** **2** → **3** and 2.31 for the reaction **1** **+** **2** → **4**) are almost two times higher than those for concerted [2+4] cycloadditions of cyclopentadiene with (*E*)-2-aryl-1-nitroethenes [2], while still being within the range typical of one-step cycloadditions [16]. Furthermore, the positive sign of the ρ constant shows that charge transfer within the transition complexes of both competing reactions occurs from the cyclopentadiene substructure towards the nitroalkene substructure. Moreover, the relationships between log *k*_A_ and log *k*_B_ and the nitroalkene global electrophilicity indexes ω are similar. In this case, however, the correlations are slightly lower.

3 $$ \log k_{\text{A}} = 3.04 \cdot \omega - 13.64\quad (R = 0.986) $$

4 $$ \log k_{\text{B}} = 2.37 \cdot \omega - 10.34\quad (R = 0.971) $$

In order to gain more insight into the nature of the transition complexes, we studied the effect of solvent polarity on the reaction kinetics. This was done for the reaction of the most electrophilic nitroalkene **2g** (Table 1). The cycloadditions **1** **+** **2g** → **3g** and **1** **+** **2g** → **4g** were found to occur more rapidly in polar than in non-polar solvents. This suggests that the transition complexes of the reactions tested are more polar than the substrates. This is consistent with the magnitude of dipole moments (μ = 7.36/8.54 D) of the respective critical structures located on the reaction potential energy hypersurface using B3LYP/6–31 g(d) calculations [5].

**Fig. 2:**
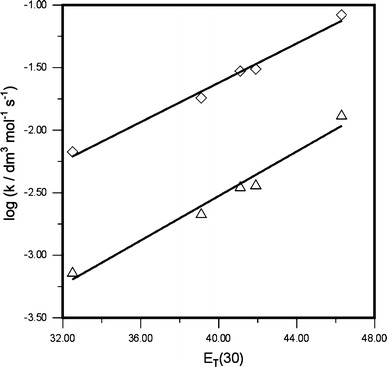
Plot of log *k* versus Reichardt–Dimroth *E*_T_(30) constants for the [4+2] cycloaddition of cyclopentadiene with (*E*)-2-(*p*-methoxycarbonylphenyl)-1-cyano-1-nitroethene (**2g**) (at 25 °C)

To obtain a quantitative description of the solvent effect on the reaction rate, we studied correlations between substrate reactivity and the solvent polarity constants such as dielectric constant ε, dipole moment μ, Berson constant Ω, and Reichardt-Dimroth *E*_T_(30) constant [18, 19]. The best linear correlations were obtained for the *E*_T_(30) constants (Eqs. 5, 6) (Fig. 2).

5 $$ \log k_{\text{A}} = 0.09 \cdot E_{\text{T}} - 6.08\quad (R = 0.987) $$

6 $$ \log k_{\text{B}} = 0.08 \cdot E_{\text{T}} - 4.76\quad (R = 0.994) $$

These data prove that solvent polarity affects the rate of **1** **+** **2g** cycloaddition to a much higher extent than that for the reaction involving (*E*)-2-phenyl-1-nitroethene [2]. However, the sensitivity constants in Eqs. 5 and 6 do not exceed 0.1 and are below the typical range observed for ionic reactions [18, 20].

Hence, the nature of the Hammett and Reichardt–Dimroth relationships suggests that the reactions **1** **+** **2** → **3** and **1** **+** **2** → **4** do not proceed according to a zwitterionic mechanism [21] (paths C and D). The values of activation parameters (Table 2) lead to similar conclusions. These parameters were determined from the rate constants of the reactions **1** **+** **2g** → **3g** and **1** **+** **2g** → **4g** at 5, 15, and 25 °C using the Eyring equation in the form:

7 $$ \log (k/T) = 10.319 + \Updelta S^{ \ne } /4.576 - \Updelta H^{ \ne } /4.576T $$

**Table 2:** Activation parameters for [4+2] cycloaddition of cyclopentadiene with (*E*)-2-(*p*-methoxycarbonylphenyl)-1-cyano-1-nitroethene (**2g**)

	Nitromethane		Chloroform	
	**1** **+** **2g** **→** **3g**	**1** **+** **2g** **→** **4g**	**1** **+** **2g** **→** **3g**	**1** **+** **2g** **→** **4g**
Δ*H* ^≠^/kJ mol^−1^	34.3	32.2	43.5	37.3
Δ*S* ^≠^/J mol^−1^ K^−1^	−165.4	−157.4	−149.9	−153.2

In particular, the activation enthalpies (Δ*H* ^≠^) were estimated from the slopes of the plots of logarithm of the ratio of *k*_A_ or *k*_B_ by the absolute temperature (*T*) versus reciprocal of the absolute temperature (1/*T*), while the entropies of activation (Δ*S* ^≠^) were determined from the intercepts of these plots. It follows from the data in Table 2 that the Δ*H* ^≠^ values for the cycloadditions **1** **+** **2g** → **3g** and **1** **+** **2g** → **4g** do not exceed 46 kJ mol^−1^. They are typical for the reactions, where the energy changes within the transition complexes resulting from breaking of π bonds existing in the substrates and formation of new σ bonds present in the cycloadducts compensate one another [16]. On the other hand, the Δ*S* ^≠^ values are high and negative (−150 to −165 J mol^−1^ K^−1^), both in the moderately polar chloroform and the highly polar nitromethane. This is typical for one-step cycloadditions proceeding through highly rigid transition complexes [22–24]. By comparison, Δ*S* ^≠^ for a two-step cycloaddition of 1,1-dimethoxy-1,3-butadiene with tetracyanoethene is −26 J mol^−1^ K^−1^ [25]. It is noticeable that the entropy changes in the transition state are much higher in our case than those for a similar reaction involving (*E*)-2-phenyl-1-nitroethene (Δ*S* ^≠^ = −102.5 to −120 J mol^−1^ K^−1^ [2]). This is probably due to the polarization and dispersion of the substrate interactions within the transition complexes, which should be much stronger in the case of more electrophilic (*E*)-2-aryl-1-cyano-1-nitroethene than in the case of less electrophilic (*E*)-2-aryl-1-nitroethenes [26, 27]. This is indirectly confirmed by the depths of the pre-reaction local minima located on the potential energy hypersurface found for the reactions involving (*E*)-2-aryl-1-cyano-1-nitroethenes [5, 9] and (*E*)-2-aryl-1-nitroethenes [1].

## Conclusion

In summary, the electrophilicity of (*E*)-2-aryl-1-cyano-1-nitroethenes is not sufficient to induce an ionic course of their [4+2] cycloaddition to cyclopentadiene. The activation parameters and the substituent and solvent effects on the reaction studied indicate its non-ionic mechanism.

## Experimental

All solvents employed for kinetic measurements were purified by standard methods [28]. Cyclopentadiene (**1**) was prepared by pyrolysis of commercially available dicyclopentadiene (Aldrich) at 180–200 °C, according to a known method [29]. Before use it was distilled under atmospheric pressure, using a 25-cm Vigreux column. (*E*)-2-Aryl-1-cyano-1-nitroethenes **2a**–**2g** were obtained by condensation of appropriate aromatic aldehydes with nitroacetonitrile, according to a reported procedure [30]. Their purity was confirmed by HPLC analyses.

Kinetic experiments were carried out in a glass reactor supplied with a thermostatically controlled jacket, magnetic stirrer, thermometer, reflux condenser, and sampling device. Liquid chromatography (HPLC) was done using a Knauer apparatus, equipped with a UV–Vis detector (λ = 254 nm). For monitoring of the reaction progress a LiChrospher 100RP column (4 × 240 mm) thermostated at 5 °C was used, and 70% methanol at a flow rate of 1.1 cm^3^ min^−1^ was applied as the eluent.

### Kinetic procedure

The overall rate constants (*k*_total_) were determined by monitoring the decrease of the HPLC peak area (*A*) corresponding to cyclopentadiene. It was found that the product composition was controlled kinetically, since the ratio of the products (γ = [**3**]/[**4**]) was constant throughout the reaction course. The kinetic experiments were carried out at 5, 15, and 25 °C up to 70–80% completion, using in each case equimolar quantities of substrates. In definite time intervals 250-mm^3^ samples were taken out of the reactor with a microsyringe and analyzed immediately by HPLC. In this way 15 series of measurements were completed. The regression analysis was carried out using MATCAD 07. It showed excellent linear relationships (*R* > 0.99) between 1/*A* and time *t* for all kinetic runs, using the second-order kinetic Eq. 8 of the form:

8 $$ 1/A = - k_{\text{total}} \cdot t + {\text{const}} $$

The *k*_total_ and γ values were then applied for calculation of the rate constants *k*_A_ and *k*_B_ according to Eqs. 9 and 10:

9 $$ k_{\text{A}} = \gamma \cdot k_{\text{total}} /(\gamma + 1) $$

10 $$ k_{\text{B}} = k_{\text{total}} /(\gamma + 1) $$

The second-order rate constants for different temperatures and solvents obtained by this method are summarized in Table 1.
